# How effective is diluted povidone-iodine in preventing periprosthetic joint infection in total joint arthroplasty (TJA)? An updated systematic review and meta-analysis

**DOI:** 10.1186/s12891-023-06548-x

**Published:** 2023-05-25

**Authors:** Mohammad-H Ebrahimzadeh, Mohammad-R Safdari, Ali Moradi, Sedighe Rastaghi, Mahla Daliri

**Affiliations:** 1 Orthopedics Research Center, Ghaem Hospital, Mashhad University of Medical Sciences, Mashhad, Iran; 2 Department of Surgery, School of Medicine, North Khorasan University of Medical Sciences, Bojnurd, Iran; 3 Department of Biostatistics, School of Public Health, Mashhad University of Medical Sciences, Mashhad, Iran

**Keywords:** Periprosthetic joint infection, Total joint arthroplasty, Diluted povidone-iodine, Wound irrigation

## Abstract

**Purpose:**

Periprosthetic joint infection (PJI) is a serious complication with total joint arthroplasty (TJA), that necessitates reoperation. Pre-closure irrigation with dilute povidone-iodine (PI) is among the preventive measures, but its efficiency is still controversial. As a result, the focus of this systematic review and meta-analysis is on the effect of dilute PI wound irrigation in the prevention of PJI following TJA.

**Methods:**

We systematically reviewed and analyzed articles that compared PI with other agents in terms of PJI rate after TJA, searching Medline, Scopus, Web of science, and Cochrane databases. A number of 13 papers including 63,950 patients in total, were finally considered in qualitative and quantitative assessments. We have also further assessed review articles.

**Results:**

In comparison with normal saline (NS), PI reduced post-operative infection rate (OR: 0.44; CI 95%: 0.34–0.56). However, there was no difference between PI and chlorhexidine (CHG) (OR: 1.61; CI 95%: 0.83–3.09)) or undetermined comparators (OR: 1.08; CI 95%: 0.67–1.76).

**Conclusion:**

PI irrigation seems an efficient preventive measure for post-operative PJI and would seem to be the most feasible for TJA protocol.

## Introduction

### Background

Peri-prosthetic joint infections (PJI) after total joint arthroplasty (TJA) is among the most serious challenges in orthopedics, worldwide [1]. Although significant attempts have been done to minimize surgical site infections (SSI) during recent decades, PJI is still estimated to occur in around 0.3 to 1.9% of total hip and knee replacements [2]. The severity and length of the treatment approach for PJI place a major load on the healthcare system, despite being uncommon [3]. Even though the World Health Organization (WHO) and the Centers for Disease Control have published two guidelines recommending intraoperative lavage with diluted Povidone Iodine (PI) [4, 5], SSI related to general surgery is distinct from PJI [6] and the beneficial effect of diluted PI for PJI prevention is still debatable [7, 8].

### Rationale

The findings of review articles on this subject are even more debatable. Two systematic reviews and meta analyses on the topic were recently published, one of which found positive results in terms of prosthesis infection prevention [9], and the other found no difference with PI irrigation [10]. In Kobayashi et al. study, the groups were sub analyzed regarding the control comparator agent. When PI was compared with normal saline (NS) irrigation, excluding studies that applied chlorhexidine (CHG) as a control or didn’t give enough information, they found that PI causes a significant drop in PJI postoperatively. However, in comparison with CHG, as an active control agent, these two solutions did not differ in their infection rate following joint replacement [8]. Kim et al., on the other hand, did not perform subgroup analysis based on the control agent, and finally found no difference in terms of infection rate between PI and non-PI application [9]. Moreover, Cacciola et al. did not conclude if diluted PI is effective in PJI avoidance or not [11]. Due to the dispute and the lack of a comprehensive systematic review that also assesses review articles (meta-research), we attempted to perform a systematic literature evaluation in the hopes of arriving at a conclusion for this critical clinical condition.

*Objectives*: The major question addressed in this systematic review is: Does the application of diluted PI irrigation before wound closure prevent PJI after TJA operations? More specifically, we aim to determine diluted PI relative effectiveness, compared with NS, CHG, and other studied control agents.

## Materials and methods

### Protocol

While conducting this systematic review and meta research, we implemented the Preferred Reporting Items for Systematic Reviews and Meta-Analysis (PRISMA) statement standards [12].

### Search strategy

The papers from databases Medline, Scopus, Web of science, and Cochrane library were screened without time limitation, using following terminology: (“povidone-iodine” OR “betadine” OR “iodo-povidone” OR “povidone”) AND (“arthroplasty” OR “TJA” OR “knee arthroplasty” OR “hip arthroplasty” OR “peri-prosthesis " OR “PJI” OR “joint arthroplasty” OR “total knee replacement OR “total hip replacement”) AND (“infection” OR “biofilm” OR “organism”). English articles were reviewed without any other filter in effect. We also looked through the citations of the articles to see whether any of the papers were relevant.

### Eligibility criteria and study selection

The present study aims to review investigations fulfill the PICOTD methodology criteria: P (Problem): post-operative PJI; I (Intervention): diluted PI wound irrigation; C (Comparison): comparison of PI and non-PI lavage groups; O (Outcomes): PJI odds ratio; T (Timing): ≥ three months’ follow-up for clinical diagnosis; D (Design): clinical trial, original prospective and retrospective articles. Additional inclusion criteria are pure PI lavage regimen, not one that includes additional solutions like Chlorhexidine Gluconate, and primary or revision arthroplasty procedure. We have also evaluated review articles (meta research), that have reviewed articles investigating dilute PI efficacy in post-operative prevention.

**Fig. 1:**
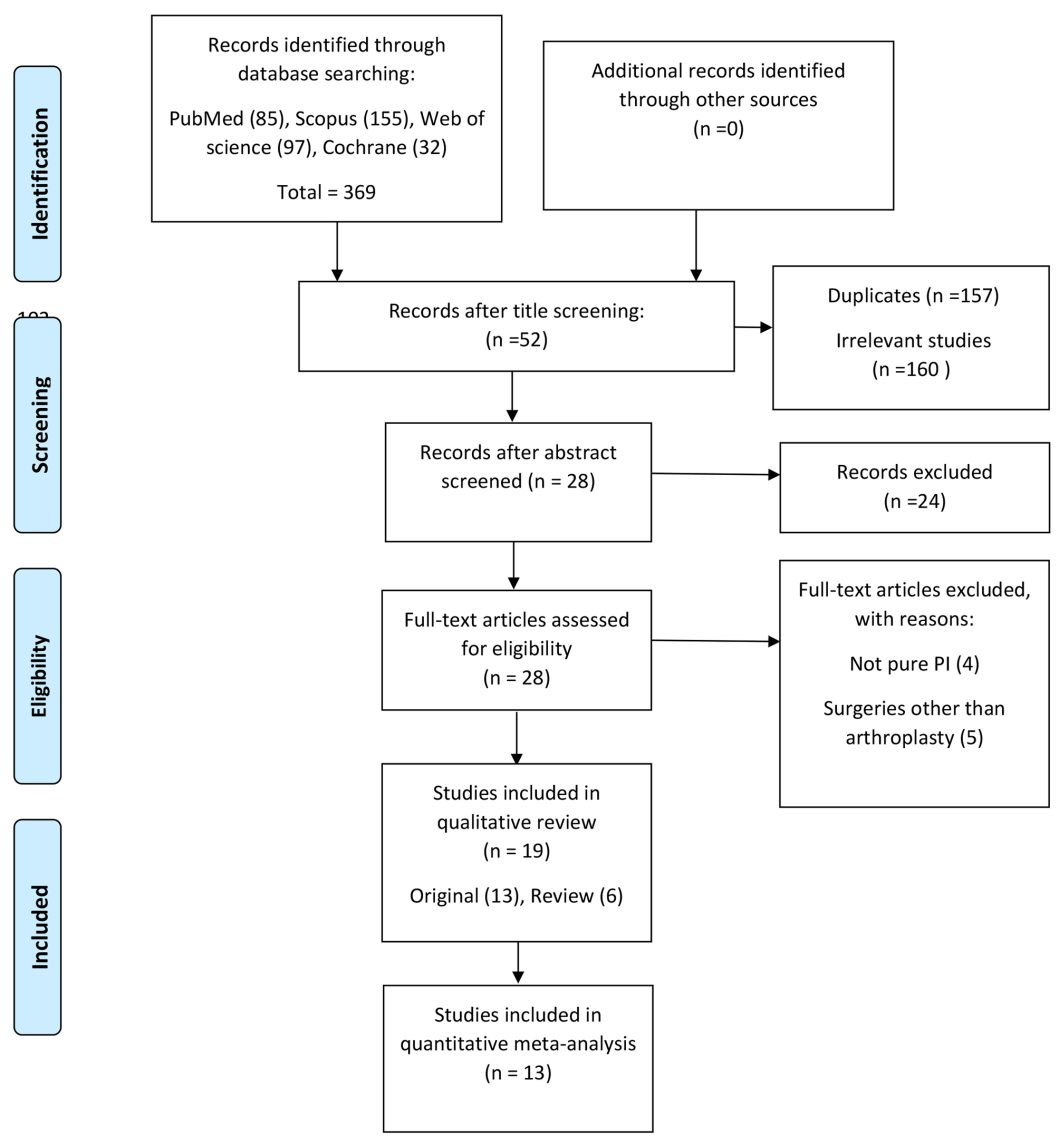
Flowchart indicating systematic search method and the number of excluded documents at each stage

The exclusion factors are defined as follows: experimental studies, biomechanical studies, case-reports, book chapters, letters to the editor, expert comments, and duplicate research. Two researchers scanned 158 articles based on title and abstract, 38 of them were read in full, and 19 papers (13 originals and 6 reviews) were included for qualitative and quantitative analysis. The search strategy flow diagram and included reports at each step are depicted in Fig. 1.

### Data extraction

Using a pre-designed Excel form, the data including first author name, study year, study design, type of surgery, sample size in case and control groups, mean age and sex ratio, follow up duration, betadine solution concentration and volume, intervention method, control group comparator, investigated infection site, infection rate in PI and control group, P value, and final conclusion were extracted.

### Quality assessment

Two reviewers evaluated each study using Methodological Index for Non-Randomized Studies (MINORS) score criteria [13] for each study methodological quality assessment. The criteria are given a score of 0 (not reported), 1 (reported but inadequate), or 2 (reported and adequate). For non-comparative research, the maximum score is 16, while for comparative studies, it is 24. The included articles’ score ranges were between 15 and 24, with the mean of 16.3.

### Statistical analysis

A meta-analysis of pooled odds ratios with 95 percent confidence intervals (CI) was created to qualitatively summarize the findings of the systematic review. Using Comprehensive meta-analysis software (Version 4 Biostat, Englewood, NJ 2022), meta-analysis was conducted. The analysis of the PJI rates between the PI and non-PI pre-closure wound irrigation groups was the primary outcome of this meta-analysis. The infection rate comparison in general, as well as the subgroup analyses for control-agent and primary vs revision arthroplasties, were conducted. Statistical significance was defined as a P value < 0.05. By calculating I^2^, the measure of treatment effect heterogeneity across studies was assessed. A random effects model was applied when conducting all the subgroup analysis. The results, pooled estimate of odds ratio, and overall summary effect of each study were displayed using Forest plots.

## Results

### Study characteristics

In total, 13 original and 6 review articles (two meta-analysis) were reviewed. Of original papers, 11 were retrospective cohort and two were clinical trials. The control comparators were NS in five studies, CHG in three researches, one sterile water, and no data available in four studies. Of all included papers, there was one clinical trial study with infection diagnosis based on culture after 48 h, and not clinical diagnosis. There were 63,950 cases of TJA (mean age: 65.69 ± 1.96, sex ratio: 55.72% female) recorded in total, with 22,765 cases undergoing PI lavage and 41,185 cases not. All of the studies included in this review were reporting THA or TKA, with 11 focusing on primary arthroplasty and two on aseptic revision arthroplasty. The further information of sample sizes, follow-up period for diagnosis of postoperative infection, and results are presented in Tables 1 and 2. Included studies were similar in terms of demographic parameters of age and sex ratio.

**Table 1:** Included studies’ characteristics

N	Author (year)	Study design	Surgery	Sample size (case + control)	Age	Sex (% female)	Follow up (months)	PI concentration	Intervention method	Control group	PJI diagnostic guideline	MINORS Score
1	Nazal et al. (2019)	RCT	Primary TJA	53 + 51			Post- operation (48 h)	0.02%	2 L of SW was placed into the splash basin, then 4 oz of 10% povidone-iodine solution was added	SW	Microbial growth after 48 h	22
2	Slullitel et al. (2019)	RSC	Primary TJA	2268 + 2268	64.5 ± 12.0	54.9	3	0.2–0.35%	22.5 mL for 1–3 min	NS	MIS and ICG	18
3	Hart et al. (2019)	RSC	Revision TJA	Hip: 231 + 946	66.5 ± 12.5	51.1	3 and 12	0.25%	1 L, 3 min followed by irrigation with normal saline solution prior to closure	NR	Infections deep to the fascia, as reported in the medical record	18
				Knee: 319 + 889	65.5 ± 11.1	52.1						
4	Hernandez et al. (2019)	RSC	Primary TJA	Hip: 912 + 3725	63.5 ± 13.7	51.2	3 and 12	0.25%	1 L, for 3 min	NR	Infections deep to the fascia, as reported in the medical record	18
				Knee: 1745 + 3489	67.4 ± 10.0	56.1						
5	Calkins et al. (2019)	RCT	Revision TJA	223 + 234	64.5 ± 9.0	50	3	0.35%	500 ml, 3 min, followed by 1 L 0.9% NaCl pulsatile lavage with skin PI painting	NS	MIS	24
6	Shohat et al. (2021)	RSC	Primary TJA	8659 + 22,672	64.7 ± 10.7	51.95	12	0.30%	PI solution was poured into the wound for approximately 3 min, after which pulsed irrigation using 1 L of sterile saline was performed	NS	International Consensus Meeting criteria 2018	18
7	Brown et al. (2012)	RSC	Primary TJA	688 + 1862	63.55	66.55	3	0.35%	500 ml, 3 min, followed by 1 L 0.9% NaCl pulsatile lavage with PI painting	NS	Following the criteria described by Lonner et al.	20
8	Driesman et al. (2020)	RSC	Primary TJA	1227 + 1159	65.14 ± 10.19	63.78	3 and 12	17.5mL of 10% povidone-iodine mixed with 500 cc of sterile saline	3 min	CHG	MIS	17
9	Frisch et al. (2017)	RSC	Primary TJA	253 + 386	65.9	60	12	< 2%	Irrigation with 0.9% saline followed by a 2-minute soak with PI which was washed out entirely before closure	CHG	MIS	15
10	Matsenko et al. (2016)	RSC	Primary TJA	1053 + 1167			12	0.35%	Soaking for 3 min with 500 mL PI solution after implantation, followed by 1 L 0.9% NaCl pulsatile lavage with skin PI painting	NR	MIS	17
11	Muwanis et al. (2022)	RSC	Primary TJA	1207 + 1511	70.8 ± 9.35	61	> 3	Betadine irrigation (6.5 mL of commercial povidone-iodine (10% solution, 100 g/L) mixed with standard 500-mL bottle of sterile saline solution	3 min	NS	International Consensus by Parvizi et al. 2014	20
12	Fleischman et al. (2018)	RSC	Primary TKA	2124 + 7665			3	Dilute PI	Irrigation	NR	CDC	NR
13	Lung et al. (2022)	Retrospective cohort	Primary TJA	206 + 204	66.3 ± 11.1	50	3	17.5 mL of 10% povidone-iodine solution with 500 mL of sterile normal saline	3 min	CHG	MIS	17

***Abbreviations:*** RCT: randomized clinical trial; RSC: retrospective cohort; PI: povidone iodine; NS: normal saline; SW: sterile water; CHG: chlorhexidine; NR: not reported; MIS: Musculoskeletal Infection Society; ICG: International Consensus Group; CDC: Centers for Disease Control

**Table 2:** Included studies’ results

N	Author (year)	Infection site	Infection rate (PI)	Infection rate (control)	P value	Other Results	Conclusion
1	Nazal et al. (2019)	120 mL aliquot sample of basin fluid was collected at incision (“preprocedure”) and closure (“postprocedure”)	0 (0%)	23 (47.9%)	< 0.001	The most common species grown were coagulase-negative Staphylococcus, Corynebacterium, and Micrococcus	Dilute PI eliminates intraoperative contamination of splash basins in TJA procedures
2	Slullitel et al. (2019)	PJI	10 (0.4%)	22 (1%)	0.033	Infected (%) per Procedure: (hip: p = 0.93), (knee: p = 0.52)/ Reoperation for all infection: p = 0.55/ Reoperation for acute infection: p = 0.76	Suggest dilute betadine lavage as an effective option of reducing acute postoperative infection since the decline in the acute infection rate was clinically meaningful.
3	Hart et al. (2019)	Hip PJI	12 (5.2%)	32 (3.4%)	0.62	No significant difference in the rate of reoperation for infection at 3 months (p = 0.58 for revision THA, and p = 0.06 for revision TKA) and at 12 months (p = 0.78 for revision THA, and p = 0.06 for revision TKA)	Following revision THA and TKA, PI wound lavage had no effect on the number of infections that required reoperation.
		Knee PJI	21 (6.6%)	34 (3.8%)	0.07		
4	Hernandez et al. (2019)	Hip PJI	5 (0.5%)	28 (0.75%)	0.93	There was no difference in the risk of septic reoperations between the groups after using the propensity score	At 3 months and 1 year after primary THA and TKA, there was no substantial reduction in the risk of infection requiring reoperation.
		Knee PJI	15(0.86%)	18 (0.51%)	0.52		
5	Calkins et al. (2019)	PJI	1 (0.4%)	8 (3.4%)	0.038	No difference in wound complications between groups (1.3% vs. 0%, P = 0.248)	A simple, safe, and effective approach to lower the incidence of acute postoperative PJI appears to be dilute betadine lavage.
6	Shohat et al. (2021)	PJI	52 (0.60%)	295 (1.30%)	< 0.001	Absolute risk reduction = 0.73% Prevent 1 PJI for every 137 TJA patients	The findings suggest the use of povidone-iodine irrigation to reduce PJI as a safe and cost-effective method.
7	Brown et al. (2012)	PJI	1 (0.15%)	18 (0.97%)	0.04	--	A low-cost, high-effective method of preventing acute postoperative infection after total joint replacement
8	Driesman et al. (2020)	PJI	14 (1.14%)	9 (0.78%)	0.48	--	While both chlorhexidine gluconate and betadine are equally effective in preventing PJI, betadine is a significantly less expensive option if sterility concerns are unfounded.
9	Frisch et al. (2017)	PJI	4 (1.6%)	3 (0.8%)	NR (> 0.05)	Nonsurgical site infections [THA: P = 0.244, TKA: P = 0.125]; superficial surgical site infection [THA: P = 0.555, TKA: P = 0.913]; and deep surgical site infection [THA: P = 0.302, TKA: P = 0.534]	We couldn’t tell the difference between chlorhexidine and dilute Betadine irrigation in terms of infection rates.
10	Matsenko et al. (2016)	PJI	4 (0.40%)	7 (0.60%)	--	--	--
11	Muwanis et al. (2022)	PJI	17 (1.4%)	45 (3%)	P < 0.05 OR: 0.45 [0.22; 0.89]	Significant reduction was seen in any infection (OR 0.45 [0.22; 0.89], P, 0.05) and SSI (OR 0.30 [0.13; 0.70], p value 0.01) with the Betadine group	Betadine compared to NS irrigation provides an inexpensive and simple method to lower any PJI and more specifically SSI in THA and TKA
12	Fleischman et al. (2018)	PJI	5 (0.2%)	46 (0.6%)	NR	--	--
13	Lung et al. (2022)	PJI	5 (1.2%)	3 (0.7%)	0.39	The 30- and 90-day emergency room readmission rate for wound complications was statistically significantly lower in all TJA patients who underwent CHG lavage.	There was no significant difference between groups in the rate of PJI requiring a return to the OR among all TJA.

***Abbreviations***: PJI: peri-prosthetic joint infection; OR: odds ratio; PI: povidone iodine; THA: total hip arthroplasty; TKA: total knee arthroplasty; TJA: total joint arthroplasty; NR: not reported

#### Individual original study results

During a retrospective cohort, dilute PI 0.35% lavage for 3 min has revealed to be efficient method for reducing acute PJI, when compared with isotonic sodium chloride solution irrigation (P = 0.04) [14]. Hernandez and Hart conducted two large cohorts on primary and revision TJA in 2019. After 3 and 12 months of follow up, they did not find any difference between infection rate between patients received PI and non-PI irrigation, neither in primary nor in revision arthroplasty [7, 15]. In contrast, there are investigations which resulted in significant decline in rate of infection with application of PI [16]. Just recently, Shohat et al. compared PI with sterile saline in a cohort with 31,331 cases, and estimated an absolute risk reduction of 0.73% when applied PI [17]. Another study with similar methodology in 2022, resulted a notable drop in any infection when arthroplasty wounds were irrigated with PI (P < 0.005) [18]. Three active comparator studies between PI and Chlorhexidine concluded in similar efficiency in infection prevention for these two agents (P = 0.53 and 0.46) [19–21]. However, a very recent retrospective analysis revealed that greater wound concerns with PI resulted in readmission to the emergency room. [21]. After performing a randomized clinical trial on 457 patients with revision TJA, Calkins et al. concluded that diluted PI lavage is a safe and beneficial approach to lower the incidence of acute postoperative PJI, compared with NS [8]. Another RCT with laboratory diagnosis of PJI 48 h after TJA, PI significantly reduced rate of positive culture results in comparison with sterile water (P < 0.001) [22].

### Individual review study results

Recently, two systematic and meta-analysis reviews have been published which came out with different conclusions [9, 10]. Kobayashi et al. indicated that PJI rate with dilute PI was notably lower than NS irrigation (P = 0.004) [9], while Kim et al. noted no difference between dilute PI and non PI (including NS) irrigation (P = 0.17) [10]. Current work includes all the studies from the 2 aforementioned review articles. Two other reviews considered betadine as an inexpensive and simple method, with PJI [23] and SSI [24] prevention potentials. One systematic review found no difference between PI lavage and non-PI agents lavage for prevention of PJI in primary and revision joint replacements [11] (Table 3).

**Table 3:** Included review articles’ characteristics and results

N	Author	Study design	Reviewed articles (N)	Study question	Sample size (PI + Non PI)	Odds ratio	95% CI	P value	Conclusion
1	Kobayashi et al. (2021)	SR and MA	8	Efficacy of diluted PI lavage for preventing PJI in primary and revision surgery	10,390 + 22,623	NS control : 0.33 CHG control: 2.17 Overall: 0.83	NS control : 0.16–0.71 CHG control: 0.97–4.87 Overall: 0.45–1.51	NS control : 0.004 CHG control: 0.06 Overall: 0.54	Diluted PI lavage is significantly better than saline solution lavage for preventing PJI.
2	Kim et al. (2020)	SR and MA	7	Does the performance of PI lavage before wound closure in TJA reduce the postoperative infection rate?	8,861 + 22,352	0.67	0.38–1.19	0.17	No differences in the overall postoperative infection rates between the PI and non-PI lavage groups before wound closure in TJA
3	Zlotnicki et al. (2021)	Review	4	The role of irrigants for prevention of PJIs					Although a role for further cocktails may have utility, dilute betadine solution remains a possible option
4	Chundamala et al. (2007)	Review	15	Determine the efficacy and risks of using povidone-iodine irrigation to prevent **surgical site infection**					Povidone-iodine irrigation is a simple and inexpensive solution with the potential to prevent surgical site infection
6	Cacciola et al. (2020)	SR	7	Current literature on the efficacy of dilute betadine in reducing PJI					Some studies found that using DPI reduces the risk of infective consequences, whereas others found no changes when DPI was utilized. More research is needed to determine the efficacy of DPI irrigation.

***Abbreviations***: SR: systematic review; MA: meta-analysis; PI: povidone iodine; NS: normal saline; CHG: chlorhexidine

#### Safety of PI versus non-PI

Hart et al. found no significant disparity in the occurrence of reoperation due to infection at both 3 and 12 months for revision total hip arthroplasty (THA) and revision total knee arthroplasty (TKA). Hernandez et al. discovered no difference in the likelihood of septic reoperations between groups after using propensity score. Calkins et al. reported that there was no significant difference in wound complications between groups. Lung et al. revealed that patients who received chlorhexidine gluconate (CHG) lavage during total joint arthroplasty (TJA) had a significantly lower rate of wound complication-related emergency room readmissions at both 30 and 90 days.

#### Quantitative results

**Fig. 2:**
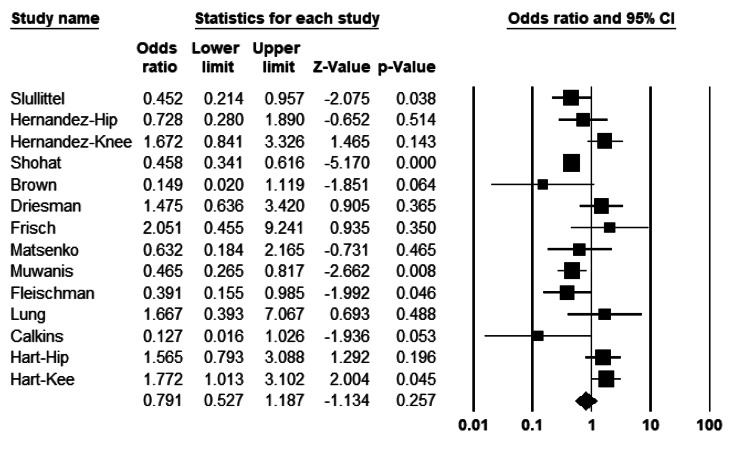
Forest plot of the postoperative infection rate between the povidone-iodine (PI) and other agents in total joint arthroplasties

In general, analysing 11 retrospective cohorts and two clinical trials comparing PI with control group, the odds ratio for PI irrigation is 0.79 (CI 95%: 0.52–1.18), which is not statistically significant (P = 0.25) (Fig. 2). The results reveal no difference between PI and non-PI irrigation. The heterogeneity index (I^2^) within groups was I^2^ 71% (P < 0.001).

##### PI versus NS

**Fig. 3:**
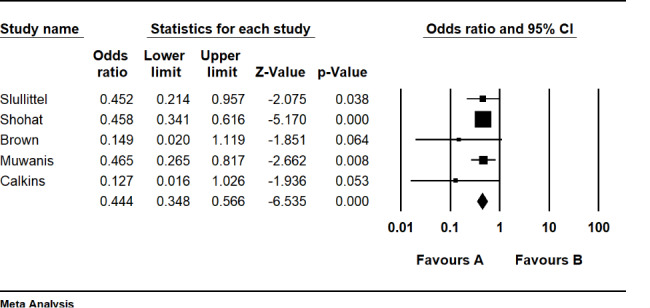
Forest plot of the postoperative infection rate between the povidone-iodine (PI) and normal saline (NS) /sterile water (SW) groups in total joint arthroplasties

Analysing four retrospective cohorts and two clinical trials, comparing PI with NS or SW, the odds ratio for PI irrigation is 0.44 (CI 95%: 0.34–0.56), which is statistically significant (P < 0.001) (Fig. 3). The results reveal notable superiority between PI and NS irrigation. The heterogeneity index (I^2^) was 0% (P = 0.63).

##### PI versus not-reported (NR)

**Fig. 4:**
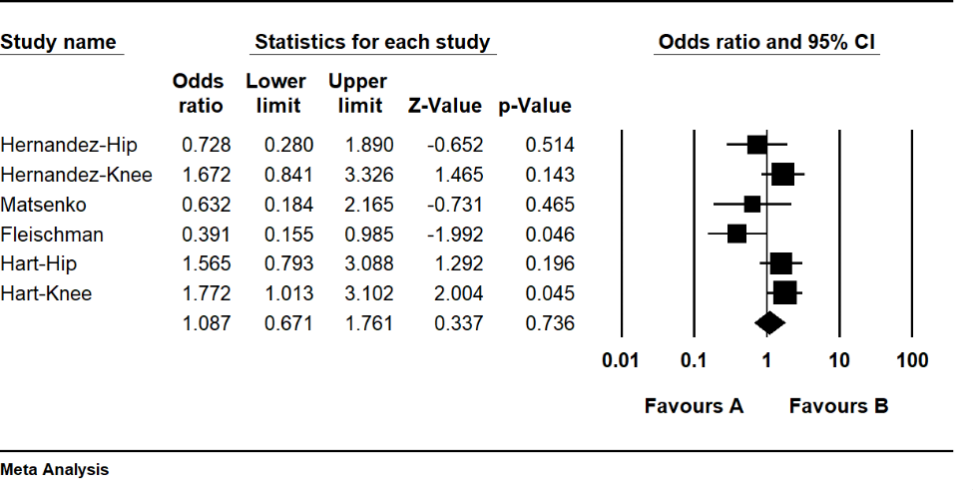
Forest plot of the postoperative infection rate between the povidone-iodine (PI) and not reported (NR) groups in total joint arthroplasties

Analysing three retrospective cohort studies comparing PI with not determined solutions, the odds ratio for PI irrigation is 1.08 (CI 95%: 0.67–1.76), which is not significant (P = 0.73) (Fig. 4). The results reveal no superiority between PI and other not-determined agents’ irrigation. The heterogeneity index (I^2^) was 54. 8% (P = 0.05).

##### PI versus chlorhexidine

**Fig. 5:**
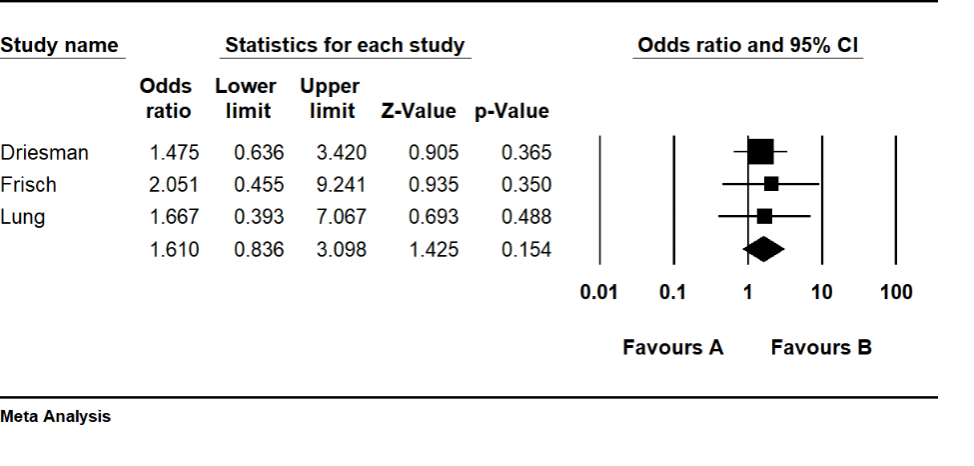
Forest plot of the postoperative infection rate between the povidone-iodine (PI) and Chlorhexidine (CHG) lavage groups in total joint arthroplasties

Analysing three retrospective cohort studies comparing PI with Chlorhexidine, the risk ratio for PI irrigation is 1.61 (CI 95%: 0.83–3.09) which is not statistically significant (P = 0.15) (Fig. 5). The results reveal no superiority between PI and Chlorhexidine irrigation. The heterogeneity index (I^2^) was 0% (P = 0.93).

##### Primary arthroplasty subgroup

**Fig. 6:**
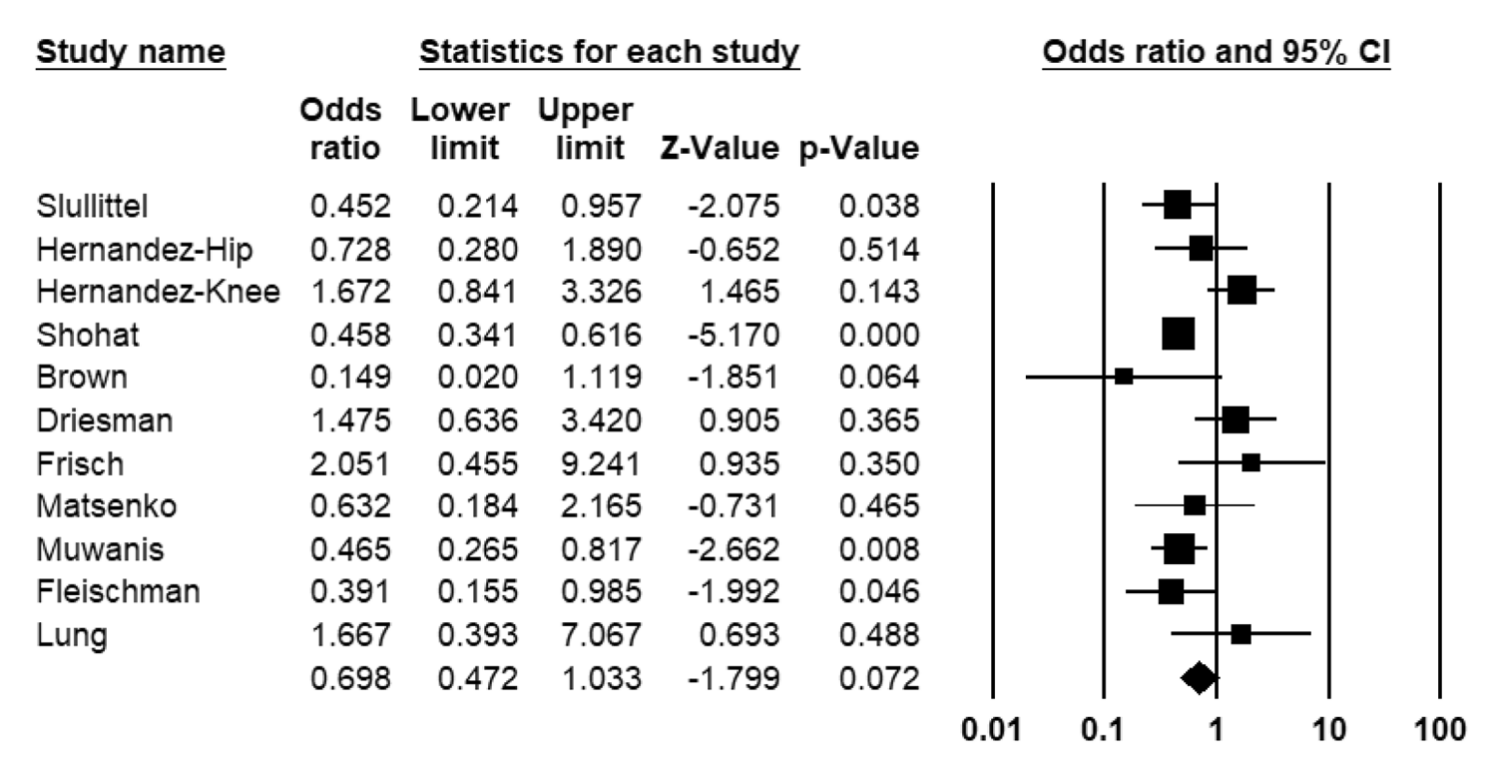
Forest plot of the postoperative infection rate between the povidone-iodine (PI) and other agents in primary total joint arthroplasties

Analysing 11 studies investigating PI vs. other agents among primary arthroplasty patients, the odds ratio for PI irrigation is 0.69 (CI 95%: 0.47–1.03), which is not significant (P = 0.072) (Fig. 6). The heterogeneity index (I^2^) was 66% (P = 0.001).

##### Revision arthroplasty subgroup

**Fig. 7:**
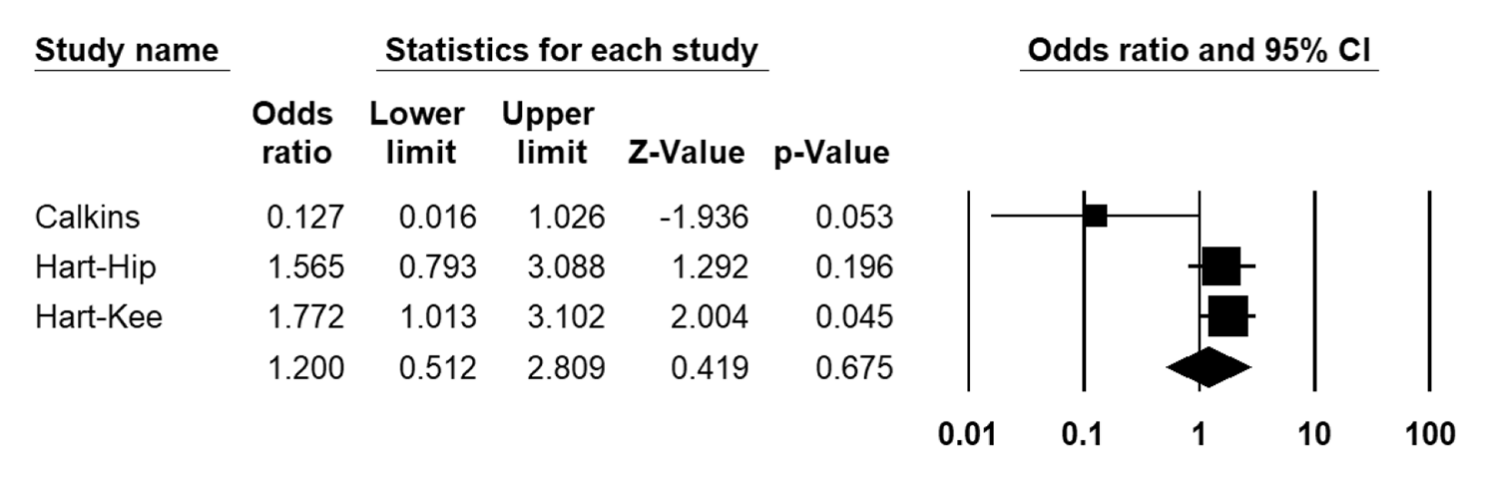
Forest plot of the postoperative infection rate between the povidone-iodine (PI) and other agents in revision total joint arthroplasties

Analysing 2 studies investigating PI vs. other agents among revision arthroplasty patients, the odds ratio for PI irrigation is 1.2 (CI 95%: 0.51–2.8), which is not significant (P = 0.67) (Fig. 7). The heterogeneity index (I2) was 65% (P = 0.06).

## Discussion

PI compound, made up of Polyvinylpyrrolidone and iodine, is a bactericidal agent works by slowly releasing free iodine, which is detrimental to bacteria [25]. When compared to other PJI preventive solutions, such as Chlorhexidine or Vancomycin, Betadine demonstrated greater bactericidal activity, killing all bacteria assayed promptly in an in vitro setting [26]. After one randomized clinical trial study, patients irrigated with PI had significantly lower positive culture rate after 48 h of arthroplasty [22]. However, there have been worries about PI irrigation safety profile. Even at low concentration, free iodine has been shown to be toxic to chondrocytes, osteoblasts, synovial cells, and bone tissues in several experiments, particularly when used more than 1 min [27–29].

Building on the most recent systematic review conducted by Kobayashi et al., our review has incorporated three additional original studies published subsequent to the Kobayashi study, along with a review of the previous reviews, to arrive at a comprehensive conclusion on this topic. The main outcome from this systematic review and meta-analysis reveals that diluted PI is superior to NS, but similar to antiseptic agents, regarding prosthesis infection prevention. Two prior meta-analyses by Kobayashi and Kim, that appears to have controversial outcomes, are consistent with our quantitative results. There is also an ongoing RCT, comparing 3.5% PI, 0.05% chlorhexidine, and sterile water, in terms of microbial growth in TJA instrument. Considering the large number of estimated participants (270 patients) and the randomized trial design, the outcomes from this study which will probably be revealed in 2023 would be helpful (https://clinicaltrials.gov/ct2/show/NCT04274517). A review article with meta-analysis, concluded that CHG was superior to PI in SSI prevention in general [30]. When skin preparation with iodine and CHG studied, iodine was superior in PJI prevention [31].

There are two recent large retrospective cohorts by one research team, in which the control group solution agents were not defined [7, 15]. Hart and Hernandez evaluated more than 10,000 individuals retrospectively with primary and revision TJA, and concluded a non-significant higher infection rate with 0.25% PI, compared with non-PI agents. These studies results are similar to the CHG control subgroup; as such, we assume they have applied an active antiseptic as the control. The PI concentration in these cohorts were lower than most of other researches using 0.35% PI, that may be hypothesis for the controversy. Of course the type of control comparators is also important in more interpretations. The clinical trial study with the highest score in quality assessment, showed that PI is an effective approach to lessen acute PJI risk [8]. Consistent with most of other studies, one another cohort in 2022, compared dilute betadine with NS on this issue, and reached to a lower rate of any infection type with betadine lavage, more notably in SSI rate [18]. CHG has been shown to reduce PJI rate more efficiently than diluted PI, albeit not significant. As such, diluted PI could be a feasible, less expensive alternative agent for CHG.

The heterogeneity among studies with not reported control agent, shows that the results of the Hart and Hernandez investigations are more in line with the CHG comparator subgroup, whereas those of the Matsenko and Fleischman studies are more in line with the NS comparator subgroup. Having stated that, we surmise that Hart and Hernandez used an antiseptic control comparator, whereas Matsenko and Fleischman used an inactive comparator (NS, SW, etc.).

Limitations: Due to the low incidence of PJI, almost all of the studies were retrospective, using different control agents and intervention approaches, resulting in heterogeneity that affected the meta-analysis results. We conducted subgroup analyses and were able to reach a homogeneous group in some cases. However, to arrive at a more robust conclusion, further well-designed prospective studies are necessary.

## Conclusion

Application of diluted PI solution for pre closure wound irrigation reduces infection rate, compared with NS lavage. In this regard, PI is probably not superior to other antiseptic agents.

## Data Availability

The data that support the findings of this study are available from Daliribm2@mums.ac.ir.

## Declarations

### Statement of the location

Orthopedics Research Center, Mashhad University of Medical Sciences (MUMS).
